# Somatic Copy Number Alterations at Oncogenic Loci Show Diverse Correlations with Gene Expression

**DOI:** 10.1038/srep19649

**Published:** 2016-01-20

**Authors:** Jason Roszik, Chang-Jiun Wu, Alan E. Siroy, Alexander J. Lazar, Michael A Davies, Scott E Woodman, Lawrence N Kwong

**Affiliations:** 1 Department of Melanoma Medical Oncology, The University of Texas MD Anderson Cancer Center, Houston, TX, 77030.; 2 Department of Genomic Medicine, The University of Texas MD Anderson Cancer Center, Houston, TX, 77030; 3 Department of Pathology, The University of Texas MD Anderson Cancer Center, Houston, TX, 77030; 4 Department of Translational Molecular Pathology, The University of Texas MD Anderson Cancer Center, Houston, TX, 77030; 5 Department of Systems Biology, The University of Texas MD Anderson Cancer Center, Houston, TX, 77030

## Abstract

Somatic copy number alterations (SCNAs) affecting oncogenic drivers have a firmly established role in promoting cancer. However, no agreed-upon standard exists for calling locus-specific amplifications and deletions in each patient sample. Here, we report the correlative analysis of copy number amplitude and length with gene expression across 6,109 samples from The Cancer Genome Atlas (TCGA) dataset across 16 cancer types. Using specificity, sensitivity, and precision-based scores, we assigned optimized amplitude and length cutoffs for nine recurrent SCNAs affecting known oncogenic drivers, using mRNA expression as a functional readout. These cutoffs captured the majority of SCNA-driven, highly-expression-altered samples. The majority of oncogenes required only amplitude cutoffs, as high amplitude samples were almost invariably focal; however, *CDKN2A* and *PTEN* uniquely required both amplitude and length cutoffs as primary predictors. For *PTEN*, these extended to downstream AKT activation. In contrast, SCNA genes located peri-telomerically or in fragile sites showed poor expression-copy number correlations. Overall, our analyses identify optimized amplitude and length cutoffs as efficient predictors of gene expression changes for specific oncogenic SCNAs, yet warn against one-size-fits-all interpretations across all loci. Our results have implications for cancer data analyses and the clinic, where copy number and mutation data are increasingly used to personalize cancer therapy.

SCNAs are commonly divided into broad and focal categories, with broad gains and losses extending up to full chromosome arms^1^,^2^,^3^. However, to date there has been no large-scale examination of the relationship between either SCNA length or amplitude to cancer driver gene expression. Consequently, in the TCGA^4^ marker papers and other studies there has been no set standard for assigning amplification and deletion status at specific loci for a given sample, possibly leading to different interpretations. Typically, each study uses a single cutoff across all loci, based on copy number amplitude and length. For example, amplification events were defined as having a log2 copy number amplitude >2.3 for bladder^5^, but >0.4 for head and neck cancer^6^. Furthermore, focal SCNAs were defined as <1 Mb for a glioblastoma analysis^7^, but as <50% of the chromosome arm for colorectal and endometrial cancer^8^,^9^. Ideally, standardized SCNA call parameters would instead ascribe optimized cutoffs for each locus, above or below which calls could be made with high sensitivity and specificity, and with consistency across studies. Information of this type will also inform the resolution of genomic data needed from clinical assays.

As a first step towards developing such standardized cutoffs for SCNAs, we have analyzed the relationship between copy number amplitude, length, and gene expression in 6,109 tumors from 16 cancer types in the TCGA dataset. This analysis identified statistically-optimized copy number amplitude and length cutoffs for nine focally-altered driver genes, with a high degree of sensitivity, specificity, and precision for predicting high-magnitude gene expression changes. *CDKN2A* and *PTEN* stand out as having unique profiles requiring SCNA length as a primary predictor of gene expression. For more than 15 other recurrently altered genes, copy number data alone is not a good predictor of gene expression changes. These results collectively represent an initial step towards standardizing functionally-relevant definitions of cancer amplification and deletion events and caution against over-interpreting single-cutoff data across all loci.

## Results

### Copy number amplitude and length in relation to gene expression between cancer types

To identify highly recurrent focal SCNAs, we first calculated the length of SCNAs (see Methods) affecting all 19,829 genes and long non-coding RNAs (lncRNAs) with matched RNA expression data across 16 cancer types for each of 6,109 samples (Table S1). As a starting point, we calculated the percentage of SCNA events <10 Mb for each gene (Fig. S1). As expected^1^,^2^, peaks were associated with known oncogenes and tumor suppressor genes. We then used this global analysis to select 17 oncogenes and tumor suppressors that are recurrently amplified or deleted and which have functional evidence of driver status^10^,^11^,^12^,^13^ (Supplementary Tables 3 and 4).

**Figure 1:**
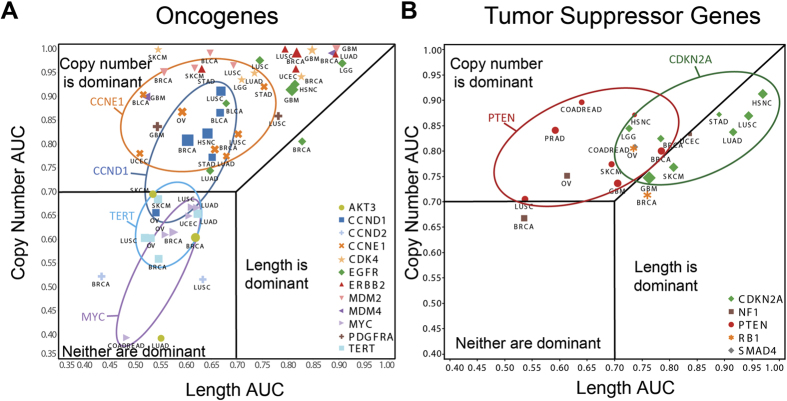
The ability of length and amplitude to independently predict high-magnitude oncogenic driver expression changes are a property of the gene locus regardless of cancer type. ROC-AUC values for 17 (**a**) oncogenes and (**b**) tumor suppressor genes measuring the ability of either SCNA copy number (y-axis) or SCNA length (x-axis) to independently predict samples with high expression changes. Selected oncogenes and tumor suppressors are circled to highlight the gene locus-centric clustering (blue: *CCND1*, orange: *CCNE1*, purple: *MYC*, cyan: *TERT*, red: *PTEN*, green: *CDKN2A)*. Only cancers are shown in which at least 15 focal SCNAs (<10 Mb) were identified for a given gene. The size of each marker correlates with the number of focal SCNAs.

For each of these 17 genes, we assessed the correlation of three parameters: SCNA copy number amplitude, SCNA length, and RNA expression. We first asked the degree to which amplitude or length can independently predict samples with high expression changes (see Methods), using receiver operating characteristic (ROC) area under the curve (AUC) calculations within each cancer type in which the gene was frequently altered (Fig. 1). We considered only samples that did not bear non-synonymous mutations for each gene. The results indicate that amplitude is a better independent predictor than length for the majority of the selected oncogenes (Fig. 1A) and tumor suppressor genes (Fig. 1B). Five genes show an exception: *CDKN2A*, *MYC*, and the peri-telomeric genes *CCND2*, *AKT3*, and *TERT*. *CDKN2A* showed a stronger dependence on length for accurately defining samples with loss of expression, while neither copy number nor length provided high AUC scores for MYC, CCND2, AKT3, and TERT.

Interestingly, gene AUCs tended to cluster together rather than cancer AUCs, especially for the outliers. *CDKN2A* has length as the dominant determinant over amplitude in 6/8 cancers, while all 16 cancer types with *MYC*, *CCND2*, *TERT*, or *AKT3* amplifications show low AUCs for both parameters. These data indicate that the locus, rather than the cancer, appears to be the dominant determinant of SCNA amplitude and length correlations with gene expression.

### Copy number amplitude and length in relation to gene expression across cancer types

The above initial analysis considered length and amplitude as independent variables and provides an overall view of each gene. To more deeply explore their interconnection, we analyzed whether interdependence exists between length and amplitude. Given the uniformity within a locus across cancer types (Fig. 1), we assayed across all 6,109 samples at once using cancer type-normalized mRNA values.

To consider both length and amplitude together, we utilized the Youden index (sensitivity + specificity – 1; which we will refer to as “performance”) which identifies cutoffs that provide optimal sensitivity and specificity from large sample sets (n > 200)^14^. We also applied the F1 score (harmonic mean of sensitivity and precision; which we will refer to as “accuracy”), which assesses how accurate that cutoff is. Youden indices and F1 scores were calculated for each gene to identify length and amplitude cutoff values that together optimally and accurately predict samples exhibiting large-magnitude gene expression changes, over a range of expression thresholds (Table 1 and Table S2).

For 7 of the 12 oncogenes tested, amplitude alone was sufficient for maximizing prediction, with length cutoffs providing no additional predictive information (Table 1). Among them, *CDK4*, *ERBB2*, and *MDM2* are most robustly predicted by amplitude with high performance and accuracy (0.8–1.0), followed by *EGFR*, *CCND1*, *CCNE1*, and *MDM4*. Strikingly, the tumor suppressors *CDKN2A* and *PTEN* required both length and amplitude cutoffs for optimal prediction (Table 1), producing good performance and accuracy. By contrast, *MYC*, *PDGFRA*, *CCND2*, *TERT*, *AKT3*, *NF1*, *RB1*, and *SMAD4* all had both low performance and accuracy, indicating a poor ability of length and amplitude to confidently separate out samples with large-magnitude expression changes.

Together, these calculations provide an evidence-based set of amplitude and length cutoffs for genes showing good performance and accuracy. For *CDK4*, *ERBB2*, *MDM2*, *MDM4*, *CCND1*, *CCNE1*, and *EGFR*, log2 amplitude cutoffs fall consistently in the 0.85 to 1.1 range (Table 1), while for *CDKN2A* and *PTEN*, log2 amplitude cutoffs are in the 0.45 to 0.6 range with a length cutoff of 10–15 Mb. We propose that these can serve as useful universal cutoffs for determining amplification and deletion status at these loci, using expression as the guiding parameter.

To illustrate these results graphically, we generated bins based on the Youden index cutoffs, with amplitude binned into high (>0.9), medium (0.58–0.9) and low (0.4–0.58), and length binned into broad (>15 Mb) and focal (<10 Mb). For clarity, we have left out samples between 10 Mb and 15 Mb, which showed a high degree of variable expression for *CDKN2A* and *PTEN*, consistent with being at the “borderline” between broad and focal SCNAs (Fig. S2). Importantly, for all genes, we considered mutant alleles separately from wild type.

**Figure 2:**
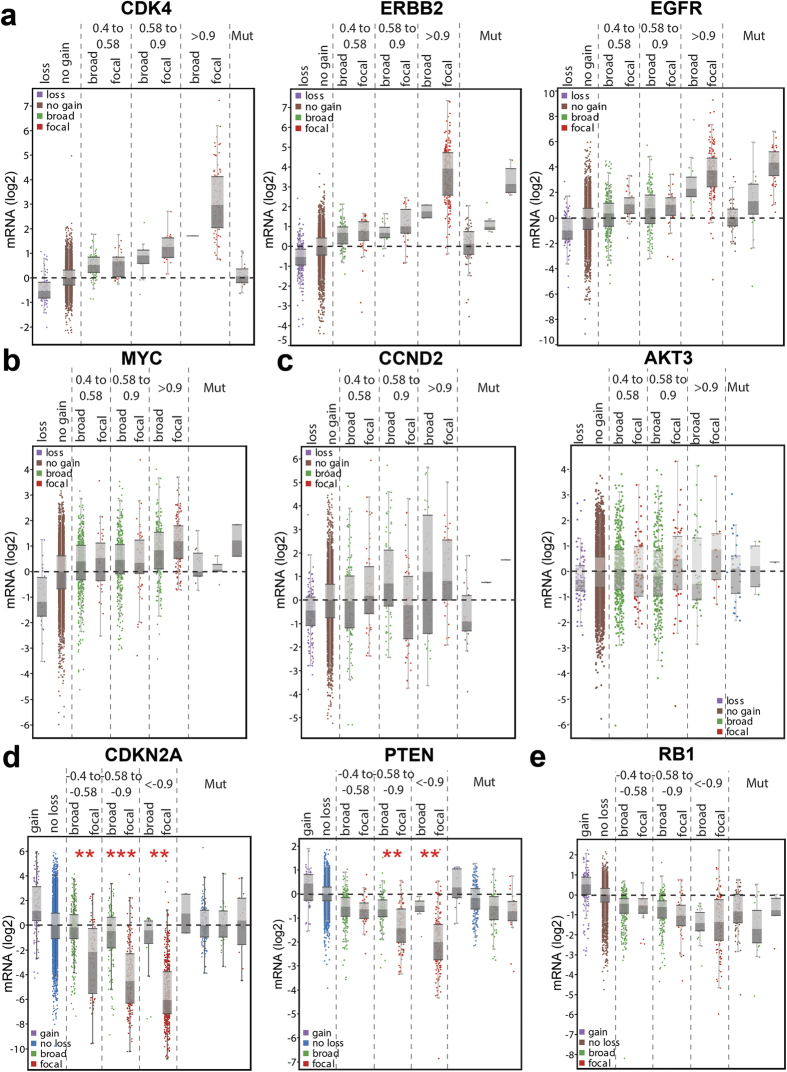
Known oncogenes and tumor suppressors display variability in correlations of expression to SCNA copy number amplitude and length. Log2 expression values (y-axis) normalized within cancer types versus binned amplitude values (x-axis) for: (**a**) three representative oncogenes whose high-expressing samples are well predicted by SCNA amplitude, (**b**) MYC, (**c**) two representative peri-telomeric genes with low predictive scores, (**d**) two tumor suppressors whose low-expressing samples are well predicted by SCNA amplitude and length together, and (**e**) a representative tumor suppressor with low predictive scores. *p < 0.05, **p < 0.01, ***p < 0.001 for broad vs focal within each bin. For each gene, any sample with non-synonymous mutations were separated out into their own group (“Mut”) to avoid the influence of mutations on CN-expression correlations.

Using this graphical output, the oncogenes showing high performance and accuracy, *CDK4*, *EGFR*, *ERRB2, CCNE1*, *MDM2*, *MDM4*, and *CCND1* (Fig. 2A and Fig. S3) show a striking pattern at log2 amplitude >0.9, with nearly all of the SCNAs being both focal (<10 Mb) and high-expressing. This indicates that amplitude is dominant in large part because high amplitude changes are already nearly always focal. Furthermore, these genes show no significant expression difference between focal amplifications and broad gains for either of the lower amplitude bins, meaning that focal but shallow amplifications do not correlate with high expression. Genes with the highest performance and accuracy scores showed the cleanest separation of the focal, high-amplitude group. We also noted that mutations in these genes had no effect on copy number-driven expression.

Among genes with low performance and accuracy, *MYC* revealed a pattern with a shallow increase in expression with copy number, and without a predominance of focal changes at high amplitude (Fig. 2B). The peri-telomeric genes *CCND2, AKT3, and TERT* revealed patterns with little if any correlation between length, copy number, and gene expression (Fig. 2C and Fig. S3). For the tumor suppressors *RB1*, *NF1*, and *SMAD4*, the high-amplitude deletions (<−0.9) are not cleanly separated from the other bins by expression (Fig. 2E and Fig. S3). These graphs collectively reveal different reasons why low prediction scores were obtained for these eight genes.

Uniquely, the tumor suppressors *CDKN2A* and *PTEN* (Fig. 2D) show robustly significant differences between focal and broad losses within amplitude bins, with the exception of the most shallow *PTEN* bin (>−0.58). These depictions reflect the key importance of length in consideration of these two genes. In other words, even when deletions are relatively shallow, samples with focal changes show a significant loss of expression. Furthermore, unlike the other 16 genes, *CDKN2A* was the only gene where mutations appeared to override SCNA changes for effects on gene expression (Fig. 2D). These results invite the careful selection of parameters when calling deletions for *CDKN2A* and *PTEN*.

To determine if the results of these analyses were affected by samples with low tumor purity, we obtained sample tumor purity and ploidy as measured by the ABSOLUTE algorithm^15^. Repeating our earlier analyses, but restricted to tumor samples with ≥50% purity, yielded identical results as the full cohort for oncogenes and for *PTEN* (Fig. S4). On the other hand, results for *CDKN2A* suggested that low purity at least partially accounts for some of the dependence of *CDKN2A* expression on length (Fig. S4): broad losses were overrepresented in the high purity group, possibly due to low purity broad losses being below our call threshold. Nevertheless, these results suggest that our SCNA amplitude and length cutoffs can identify highly expression-changed samples regardless of sample purity.

### Expression alone is not sufficient to discriminate drivers from passengers

**Figure 3:**
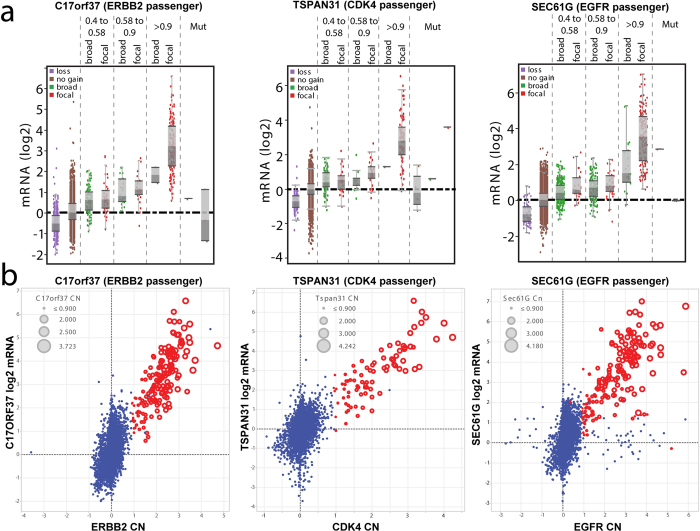
Passenger genes adjacent to known oncogenes exhibit expression profiles similar to the oncogenes. (**a**) Amplitude-binned expression profiles of three representative “passenger” genes. (**b**) Overlap between high-expressing, high-amplitude samples between the “passenger” gene and the adjacent oncogene. Samples highlighted in red are high amplitude (>0.9) for both the “passenger” and its adjacent oncogene.

Often, the correlation of expression to SCNA amplitude is presented as evidence of oncogenic status. To assess the strength of such evidence, we asked whether SCNA-driven expression patterns alone are able to discriminate between drivers and passengers within amplicons. We analyzed 20 such potential passenger genes located within our identified amplicons (Table S3) and found that more than half exhibit SCNA-driven expression patterns largely indistinguishable from those of bona fide drivers. We highlight examples of three “passengers” that are directly adjacent to known driver oncogenes: *C17orf37* (adjacent to *ERBB2*), *TSPAN31* (adjacent to *CDK4*), and *SEC61G* (adjacent to *EGFR*). In each case, the binned pan-cancer profiles of the “passengers” strongly resemble that of the adjacent driver oncogene, with high-level amplifications correlating with high expression (Fig. 3A, compare with Fig. 2A). Furthermore, when looking at all pan-cancer amplification events that include both the “passenger” and the driver, the “passenger” shows co-overexpression in nearly every instance (Fig. 3B). These findings are consistent with a recent report showing a strong gene dosage dependence throughout the genome^16^. Overall, these results imply that expression patterns alone are not sufficient to distinguish drivers from potential passengers within a given amplicon, and conversely leaves open the possibility that some of these “passengers” may also contribute oncogenic functions.

### Fragile site genes

**Figure 4:**
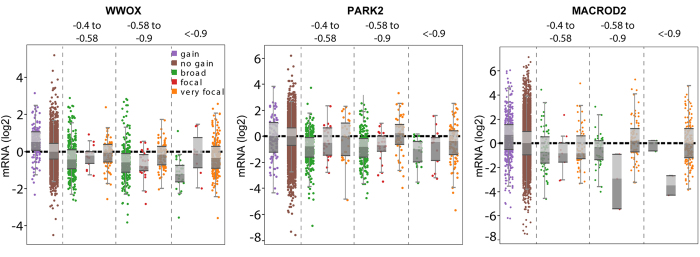
Amplitude-binned expression profiles of three representative fragile site genes. Log2 expression values (y-axis) normalized within cancer types versus binned amplitude values (x-axis).

We also identified a number of frequently deleted “fragile site” genes^17^ (Supplementary Tables 3 and 4). These are believed to be common DNA breakage points, and there is contention as to how many of these affect true tumor suppressors^18^,^19^. On the global focal SCNA plot (Fig. S1), these appear as sudden “jumps” rather than gradual peaks, indicative of a relatively large number of deletions consistently spanning only one or very few genes. We therefore asked whether expression correlation analyses can provide any insight. We analyzed four fragile site genes that are deleted at relatively high frequency across all cancer types: *A2BP1* (7%), *PARK2* (5%), *WWOX* (7%), and *MACROD2* (6%), of which the first three have documented functional evidence of tumor suppressor activity^20^,^21^,^22^,^23^. *A2BP1* expression was undetectable in the majority of samples, and was not studied further. For the other three genes, focal deletions did not show a consistent correlation with expression compared to euploid samples, regardless if the focal deletion spanned only the one gene (“very focal”) or multiple genes (“focal”) (Fig. 4). Similar results were obtained for *LRP1B*, *FHIT*, *FAM190A*, and *PDE4D* (not shown). This data suggests that focal deletions at these loci may affect the function of the genes in ways that are not necessarily correlated with mRNA expression level.

### Detailed analysis of PTEN

**Figure 5:**
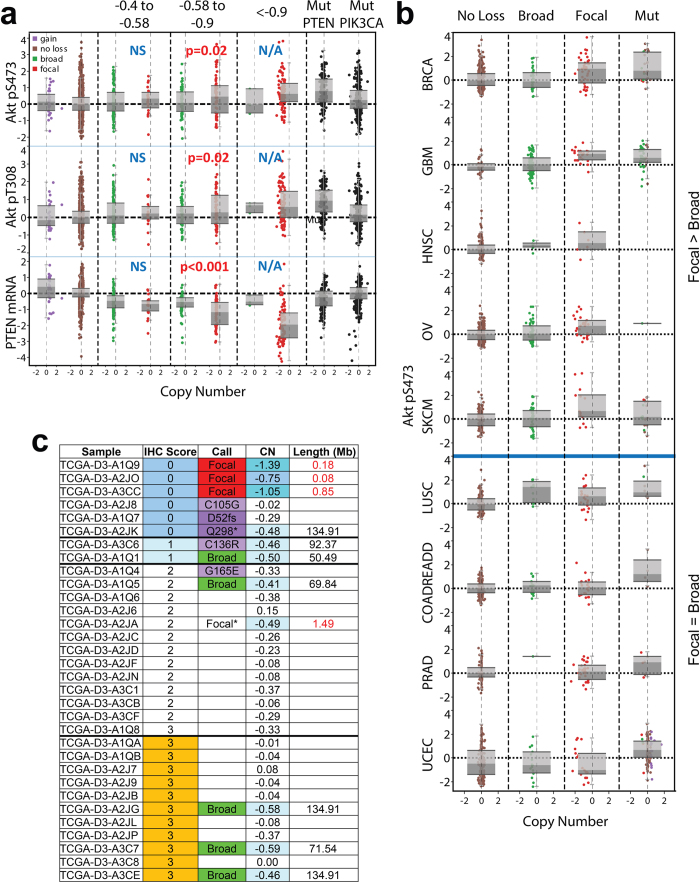
Akt activation shows a similar pattern to *PTEN* loss in the correlation of expression to SCNA amplitude and length. (**a**) Binned chart comparing phosphorylated Akt protein at two residues from RPPA data along with *PTEN* mRNA. Both PTEN and PIK3CA mutants are separated out as an otherwise confounding factor. P-values compare broad and focal within each bin. (**b**) A simplified breakdown of pAkt levels by cancer type. (**c**) Immunohistochemical analysis of the correlation between Pten protein levels and *PTEN* focal loss, broad loss, or mutation status on a subset of TCGA samples.

Our analyses indicated that the RNA expression of *PTEN* SCNAs correlates with deletion length. Given that PTEN loss is an inclusion criteria in several clinical trials (e.g. NCT01430572, NCT02286687), we selected it for deeper analysis. We therefore leveraged samples in the TCGA cohorts with reverse phase protein array (RPPA) data to determine the relationship between SCNA properties and downstream signaling effects. Given that PTEN loss has been an inclusion criteria for patient participation in several clinical trials, we selected it for deeper analysis. We leveraged samples in the TCGA cohorts with reverse phase protein array (RPPA) data to determine the relationship between SCNA properties and downstream signaling effects. Given that *PTEN* loss is a primary driver of Akt activation (assessed by phosphorylation of Akt at Ser473 and Thr308), and that each of these proteins is well represented in the TCGA RPPA set, we focused on the relationship between broad versus focal *PTEN* deletion and downstream signaling through Akt. We observed that focal but not broad *PTEN* loss correlated with both significantly decreased PTEN protein levels and increased pAkt protein expression, supporting functional impact for focal deletions. Notably, focal *PTEN*-correlated activation of pAkt was only seen at bins below −0.58, consistent with our earlier *PTEN* mRNA analyses (Fig. 5A and Fig. S5). To further probe this relationship, we analyzed *PTEN* in nine individual cancer types characterized by focal *PTEN* loss. The majority of these cancers reflect the pattern of focal PTEN loss/mutation-specific pAkt elevation, though there is also some cancer-type specificity (Fig. 5B) despite *PTEN* expression levels being consistently decreased in focal deletions (Fig. S5). We note that urothelial cancer (UCEC) may be a unique outlier due to an extraordinarily high level of *PTEN* mutations (65%). Overall, these results suggest that for the functional readout of Akt activation, *PTEN* calls are still dependent on both deletion length and amplitude.

In order to further validate these findings, we evaluated Pten protein expression by immunohistochemistry (IHC) on a set (n = 32) of melanomas included in the TCGA analysis. The IHC staining confirmed that complete loss of Pten protein expression, which has previously been shown to correlate with increased pAkt^24^, is seen only when the DNA locus is focally deleted at high-medium amplitude (e.g. A2JO) or when mutated (e.g. A2J8). Samples with broad deletions (e.g. A3C7) or very shallow focal deletions (e.g. A2JA) in *PTEN* were overall indistinguishable from euploid tumors (Fig. 5C and Fig. S6). These results emphasize the need to incorporate both length and copy number amplitude when scoring patient samples for *PTEN* loss using only next generation sequencing analysis, as focal and broad losses likely translate into different downstream pathway activation statuses. For example, a trial using biallelic *PTEN* deletion from aCGH data as an inclusion criteria potentially could identify additional patients by including medium-amplitude (0.6–0.9), but focal *PTEN* deletions^25^.

## Discussion

In analyzing copy number data for both computational and clinical needs, a critical question is whether a particular locus should be called an amplification/deletion or not for each patient sample. Indeed, under- or overestimating potentially functional SCNAs in populations can lead to a less clear understanding of a cancer’s genomic landscape. To begin to address this, we have asked whether using expression data as a readout can reveal optimized cutoffs for known oncogenic loci, using 6,109 samples from the TCGA dataset. We discovered that these loci have diverse correlations between copy number attributes and gene expression, suggesting that different loci require different criteria for calling its SCNA status.

By statistically assessing samples with high-magnitude changes of expression, we were able to assign optimized cutoffs for copy number amplitude and length for nine key oncogenic loci. Importantly, our assessments indicate that for seven of these genes, copy number-based cutoffs capture the majority of high-magnitude expression-changed samples. For these genes, this bolsters the use of both expression as a relevant readout and copy number length and amplitude as appropriate cutoff parameters. These results have implications for the assessment of large-scale copy number data. For example, the expression-optimized cutoffs suggest that CCND1 amplifications are overestimated in TCGA head and neck cancer which uses a low threshold (31% vs 18% prevalence) and underestimated in TCGA bladder cancer which uses a high threshold (6% vs 8% prevalence). These differences could have important ramifications when considering the use of targeted therapies for both populations and for specific patients.

More broadly, we found that we can categorize 20 assayed genes into: i) oncogenes where a high log2 amplitude (>~0.9) alone identifies the majority of focal, high-expressing samples with good performance and accuracy (*CCND1*, *CCNE1*, *CDK4*, *EGFR*, *ERBB2*, *MDM2*, *MDM4*); ii) oncogenes and tumor suppressors showing less readily-separable expression correlations, albeit for different reasons (*MYC*, *AKT3*, *CCND2*, *TERT*, *NF1*, *RB1*, *SMAD4*, *PDGFRA*, fragile-site genes); and iii) tumor suppressors with dependence on both length and amplitude (*CDKN2A*, *PTEN*). In the case of *PTEN*, these observations extended to the downstream functional output of Akt phosphorylation, consistent with *PTEN’s* role as a key regulator of the oncogenic PI3K signaling pathway. Overall, these results strongly caution against a one-size-fits-all approach to identifying functionally relevant SCNAs.

Clinically, these findings are likely to have important implications, as the clinical use of next generation sequencing (NGS) panels is expanding rapidly, and particularly as personalized therapies are recommended based on genomic data. For example, *PTEN* status has been evaluated as a mechanism of therapeutic resistance^26^,^27^,^28^,^29^, and has been used as an inclusion criteria in several clinical trials, including use of array comparative genomic hybridization data^25^. SCNAs of oncogenic receptor tyrosine kinases (RTKs) have also been a focus of patient selection and drug development for tyrosine kinase inhibitors (TKIs)^30^,^31^, and alterations in *CDKN2A*, *CCND1*, and *CDK4* are being investigated as predictors of outcomes with cyclin-dependent kinase (CDK) inhibitors^32^,^33^,^34^. Thus, a clinician, for whom copy number and mutation data are increasingly readily available, can make use of the optimized cutoffs to maximize the utility of the available data. Additional information from therapeutically relevant but less frequently focally altered drivers, such as *ARID1A*^35^ or *MET*^36^, awaits the accumulation of even larger datasets to provide robust statistical characterization.

We also emphasize that the high performance and accuracy scores of our tests for *CDK4*, *ERBB2*, *MDM2*, *MDM4*, *EGFR*, *CDKN2A*, and *PTEN* indicate that copy number changes drive the majority of expression differences at these seven loci. Other processes such as methylation and miRNA suppression, however, remain important in identifying additional aberrant samples. The remaining assayed genes likely have additional strong drivers of expression, including epigenetic regulation, miRNA suppression, upstream transcription factors, feedback loops, promoter mutations in the case of *TERT*^37^, or enhancer-targeted rearrangements such as seen with TERT^38^ and MYC^39^. In particular, our results for peri-telomeric and fragile site genes suggest caution in assigning amplification/deletion status at these loci to a particular tumor^40^,^41^,^42^, as the functional impact of such amplifications may not be straightforward.

We also confirmed that expression alone is not sufficient to discriminate between known drivers and passengers within a focal amplicon. Relatedly, we observed that broad gains and losses tend to affect the expression of most genes in the region as well, consistent with previous reports^16^,^43^. In considering such broad versus focal SCNAs, we note that virtually all of the high-magnitude expression changes across our assayed genes were coincident with focal rather than broad changes (Fig. 2). Consistent with this, broad loss of *PTEN* at any amplitude did not correlate with consistent activation of AKT by phosphorylation, but rather focal SCNAs. Thus, focal SCNAs may experience a different selective pressure than broad SCNAs, aimed at generating large expression changes. Therefore, claims that specific oncogenes or tumor suppressors are targeted by either broad or focal SCNAs must be rigorously supported by functional assays with physiologically-relevant gene expression changes commensurate with patient samples, for example with *PIP4K2B*^44^, *GOLPH3*^45^, or *SOX2*^46^.

As the number of assayed tumor samples accumulates through TCGA, ICGC (International Cancer Genome Consortium), and other large-scale efforts, and as the sophistication of analyses grows, we anticipate a heightened need for rigorous definitions of oncogenic states with an eye towards clinical applicability. This study provides a first step by emphasizing the diversity of gene expression changes modulated by SCNAs, meaning that driver genes will require individual parameters for functional genotyping calls affecting downstream therapeutic decisions. Multiplatform analyses such as the ones analyzed in this study, further refined by the addition of future samples, hold promise for enabling such precision clinical assessments.

## Methods

### TCGA data sources

Copy number (SNP array), mRNA expression (RNA sequencing), and protein expression (reverse-phase protein array, RPPA) data were downloaded from public TCGA repositories (https://tcga-data.nci.nih.gov and http://gdac.broadinstitute.org; primary dates of access, 4/1/2014–5/1/2014). Copy number (SNP array) data was obtained from the “minus_germline_cnv” segmented SCNA data. In our analyses, we used the following 16 TCGA disease types: Bladder urothelial carcinoma (BLCA), Breast invasive carcinoma (BRCA), Colon and rectum adenocarcinoma (COAD, READ), Glioblastoma multiforme (GBM), Head and neck squamous cell carcinoma (HNSC), Kidney renal clear cell carcinoma (KIRC), Kidney renal papillary cell carcinoma (KIRP), Brain lower grade glioma (LGG), Lung adenocarcinoma (LUAD), Lung squamous cell carcinoma (LUSC), Ovarian serous cystadenocarcinoma (OV), Prostate adenocarcinoma (RPAD), Skin cutaneous melanoma (SKCM), Stomach adenocarcinoma (STAD), Thyroid carcinoma (THCA), and Uterine corpus endometrial carcinoma (UCEC). ABSOLUTE purity and ploidy values were obtained from the PANCAN12 project^47^.

### Calculation of SCNA lengths

We developed an R program^48^ which was used to determine the length of amplification or deletion for every gene for all samples in 16 cancers. For each gene, we assigned its amplitude as the maximum change at any point across its locus in order to include SCNAs that affect only part of the gene. The program then uses the following algorithm. Starts and ends of SCNAs were defined at or above ±0.4 log2 copy number amplitude, with a new length calculation being initiated if the amplitude incremented by more than ±0.15. Thus, to determine the SCNA length, the program starts at each gene and probes in each direction until either a >±0.4 amplitude is encountered. Amplitude changes >±0.15 are considered separate deletions/amplifications, unless the amplitude is very high (>±0.9). At these higher amplitudes above ±0.9, SCNA lengths were continuous no matter how high the amplitude reached. Varying the cutoffs in the calculations by ±0.05 produced no effect on the relationships between SCNA length and gene expression. This method purposely produces more conservative lengths than the ziggurat deconstruction employed by GISTIC 2.0^1^, as we were looking for rate-limiting factors.

### Selection of the 17 Oncogenes and Tumor Suppressors

Using the genome-wide SCNA length readout, we calculated the percentage of events that were <10 Mb length and >±0.4 log2 amplitude for every gene for all 6,109 samples. We next identified amplification peaks. From the full list of 19,829 genes, we identified 66 genes (Supplementary Table 3) that had both a focal SCNA rate >2.5% and which were located in focal SCNA “peaks” (see Supplementary Fig. 1) defined as a >1.5-fold increase in amplitude compared to the median amplitude of adjacent DNA within 5 Mb in both directions. This methodology excluded genes within 5 Mb of telomeres. We chose 2.5% frequency as a cutoff to ensure sufficient statistical power for downstream calculations. These 66 genes represented 14 segments of contiguous DNA. We then identified genes for which functional validation data was readily available in the literature. This resulted in the selection of 9 oncogenes: *MDM4, PDGFRA, EGFR, MYC, CCND1, CDK4, MDM2, ERBB2*, and *CCNE1*. The same methodology was applied to deleted genes, identifying 66 genes in 39 segments (Supplementary Table 4). From this, we selected 5 tumor suppressor genes supported by functional validation in the literature and which did not exhibit characteristics of fragile site genes^18^: *CDKN2A, PTEN, RB1, NF1*, and *SMAD4*. We further selected *CCND2*, *AKT3*, and *TERT* for analysis as frequently amplified genes located near telomeres with known oncogenic function. The SCNA amplitude of each gene is assessed as the maximal amplitude within the gene locus.

### Receiver Operating Characteristic (ROC) and Prediction Score Calculations

To generate mRNA values suitable for comparison across cancer types, we normalized within each cancer to the median value of samples “euploid” (log2 amplitude <0.4) and wild type at that locus. We also excluded samples with mutations in the gene of interest, to avoid this potential confounding factor. We then developed a VisualBasic program for Excel which assigns true or false values to mRNA expression, with a specified threshold over the cancer-specific euploid median assigned as “true” overexpressing. Varying these thresholds, as well as including a second threshold only below which were assigned false values as a means of censoring “borderline” samples, did not affect the overall conclusions of the analyses (Supplementary Table 2). The program then measures the sensitivity and specificity values for copy number amplitude (high to low) or length (low to high), and calculates the area under the curve. For Youden indices, the program generates a table of length x amplitude cutoffs, set at 1 Mb intervals for length or 0.5 intervals for amplitude, and calculates (sensitivity + specificity - 1) for each possible pairwise combination. The pair that gives the highest value is then identified for each gene. The F1 score for the best Youden index is calculated as the harmonic mean of sensitivity and precision. The Youden index is on a range of −1 to +1; the F1 score is on a range of 0 to +1. For these calculations, only samples with SCNAs (i.e. >0.4 amplitude) at that locus are considered, between 250–1000 samples per gene.

### Display of mRNA values

All graphs were generated using the Tableau software (http://www.tableau.com).

### Immunohistochemistry

Immunohistochemistry was performed as previously described^24^,^49^. Briefly, the PTEN monoclonal antibody 6H2.1 (Cascade Bioscience, Winchester, MA, USA) was used and results were scored as positive (any reactivity, graded on an increasing scale of 1–3) or negative (<10% of tumor cells with any immunoreactivity; grade of 0).

## Additional Information

**How to cite this article**: Roszik, J. *et al.* Somatic Copy Number Alterations at Oncogenic Loci Show Diverse Correlations with Gene Expression. *Sci. Rep.* **6**, 19649; doi: 10.1038/srep19649 (2016).

## Supplementary Material

Supplementary Information

## Figures and Tables

**Table 1:** Optimal length and amplitude cutoffs when considered together, for 17 oncogenes and tumor suppressors. Best scores are shown from several tested expression thresholds (see Supplemental Table 2).

Oncogenes	Length cutoff (Mb)	Amplitude cutoff (log2)	Youden Index “performance”	F1 Score “accuracy”
**CDK4**	NC	**0.9**	**0.910**	**0.914**
**ERBB2**	NC	**0.95**	**0.893**	**0.935**
**MDM2**	NC	**1.05**	**0.829**	**0.851**
**MDM4**	NC	**0.9**	**0.772**	**0.568**
**EGFR**	NC	**1.1**	**0.650**	**0.782**
**CCND1**	NC	**1.1**	**0.564**	**0.756**
**CCNE1**	NC	**0.85**	**0.452**	**0.669**
**PDGFRA**	N/A	N/A	0.492	0.574
**MYC**	N/A	N/A	0.324	0.229
**CCND2**	N/A	N/A	0.317	0.415
**AKT3**	N/A	N/A	0.225	0.279
**TERT**	N/A	N/A	0.142	0.342
**Tumor Suppressor Genes**	**Length cutoff (Mb)**	**Amplitude cutoff (log2)**	**Youden Index “performance”**	**F1 Score “accuracy”**
**CDKN2A**^**a**^	**11**	**0.45**	**0.676**	**0.868**
**PTEN**^**b**^	**15**	**0.6**	**0.603**	**0.748**
**SMAD4**	N/A	N/A	0.433	0.444
**NF1**	N/A	N/A	0.384	0.542
**RB1**	N/A	N/A	0.317	0.559

NC = no cutoff, as all length cutoffs equaled or underperformed against no cutoff at all.

N/A = not applicable due to low Youden index and F1 Score.

a = does not include samples with CDKN2A mutations.

b = does not include samples with deletions below 0.08 Mb length, due to apparent artifacts.
