# Supplementary material for: Somatic Copy Number Alterations at Oncogenic Loci Show Diverse Correlations with Gene Expression
Source: Sci Rep. 2016 Jan 20;6:19649. doi: 10.1038/srep19649 (PMC4726397; doi:10.1038/srep19649)
Supplement: Supplementary Information [file srep19649-s1.pdf]

Somatic Copy Number Alterations at Oncogenic Loci Show Diverse Correlations with Gene Expression

Jason Roszik, Chang-Jiun Wu, Alan E. Siroy, Alexander J. Lazar, Michael A Davies, Scott E Woodman, Lawrence N Kwong

Supplementary Figures and Tables.

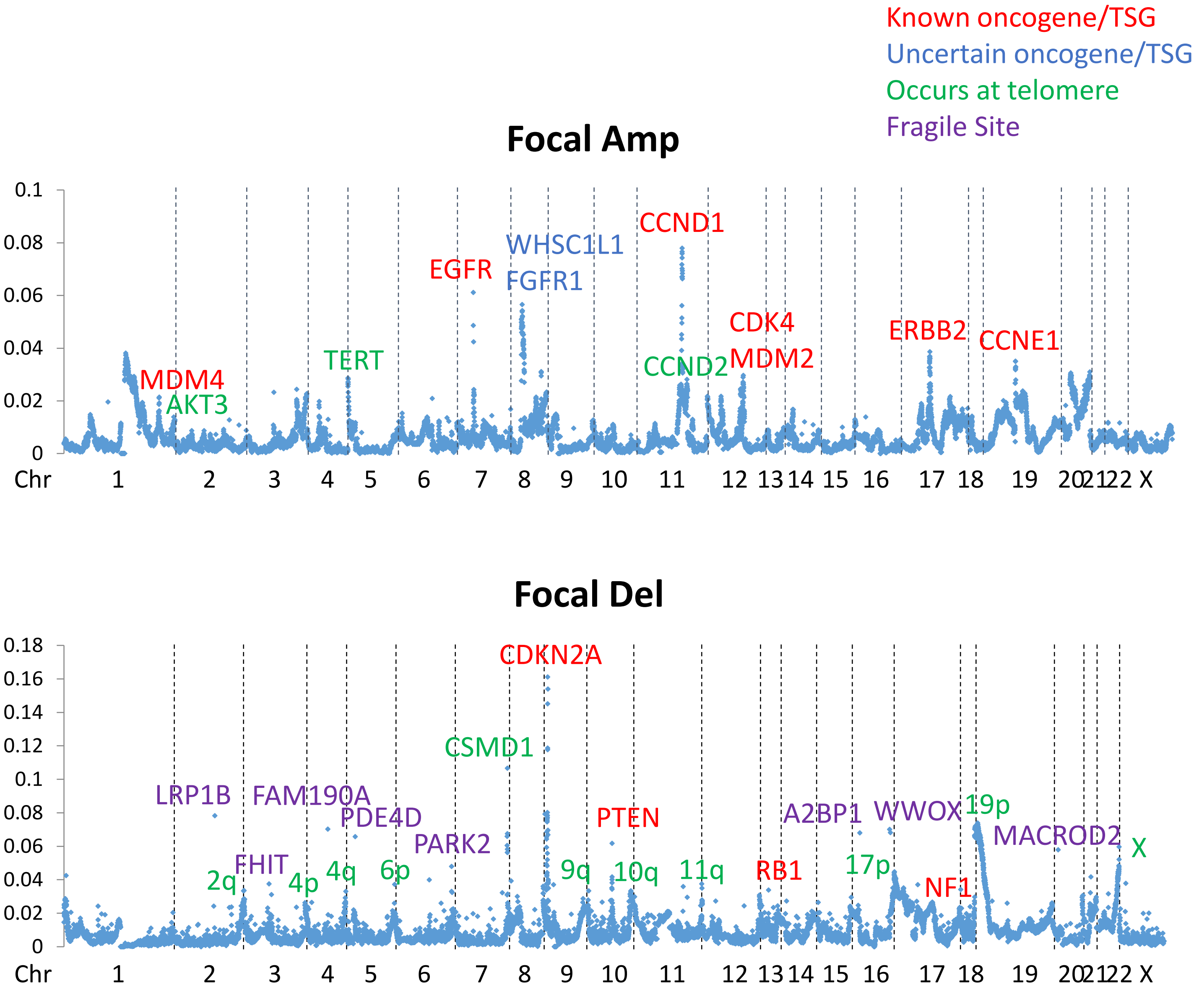

Figure S1: Global patterns of focal (<10Mb), medium/high copy number (>0.6) SCNAs across all cancers. Y axis indicates percentage of all 6109 TCGA samples. X axis represents gene loci from chromosome 1 to X. Selected oncogenes, tumor suppressors, fragile site genes, and telomeric sequences are shown above their corresponding peaks.

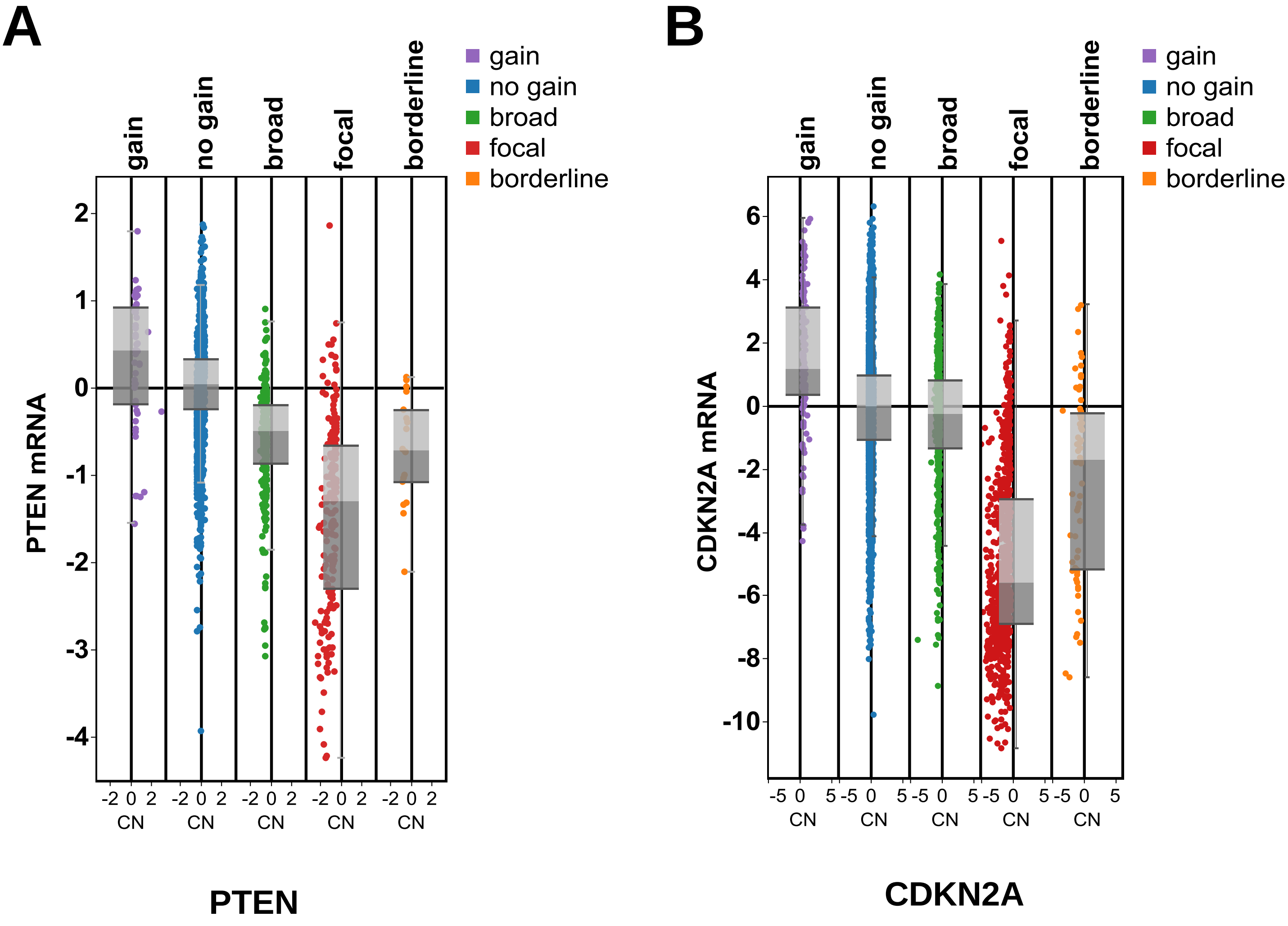

Figure S2: Analysis of borderline deletions (10-15Mb).

The mRNA expression of borderline PTEN deletions is significantly different from focal ($p<0.05$) but not from broad deletions (A). Similarly, mRNA expression of borderline CDKN2A deletions is significantly different from focal ($p<0.01$) but not from broad deletions (B).

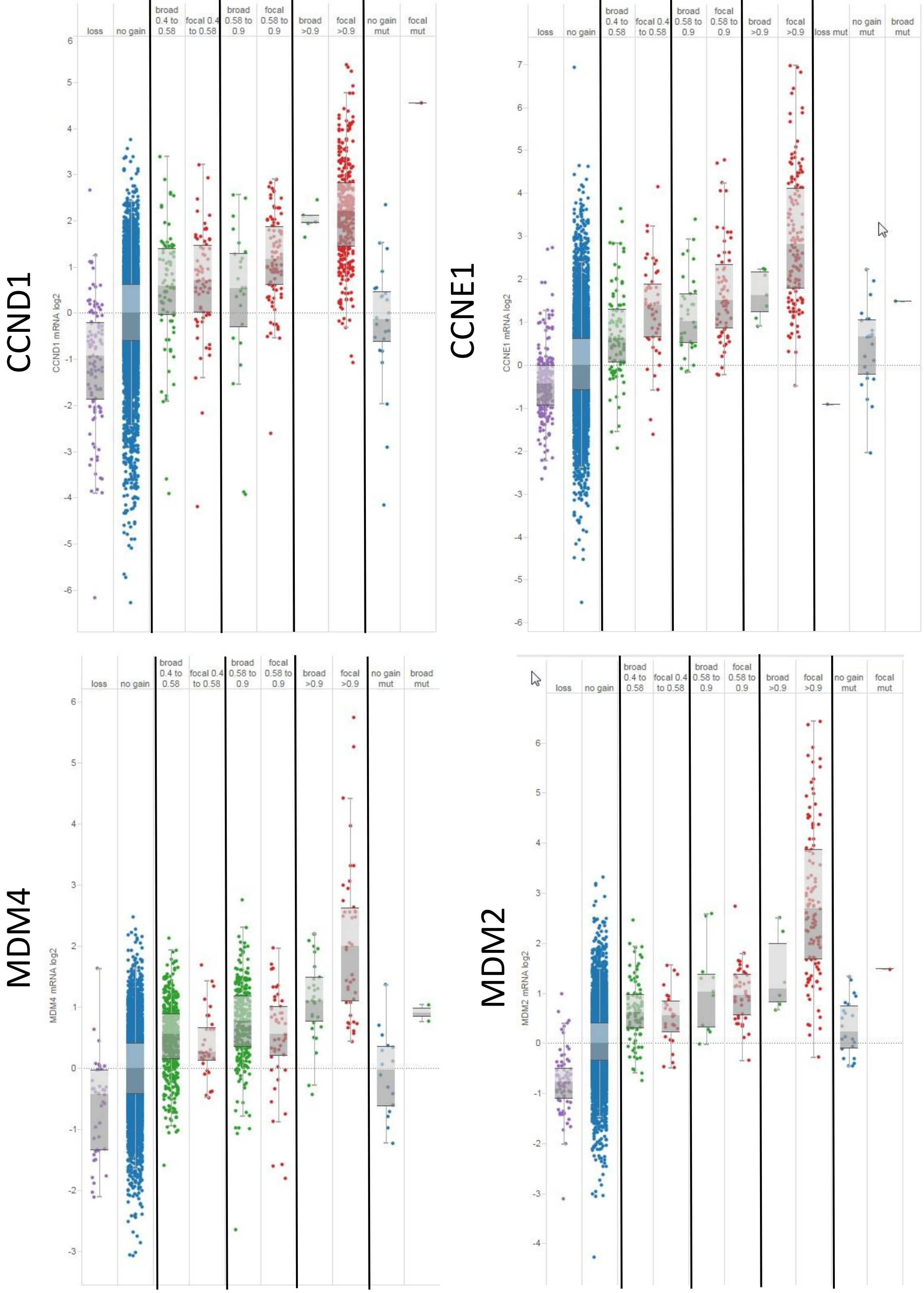

Figure S3: Additional binning graphs of genes not depicted in Fig. 2.

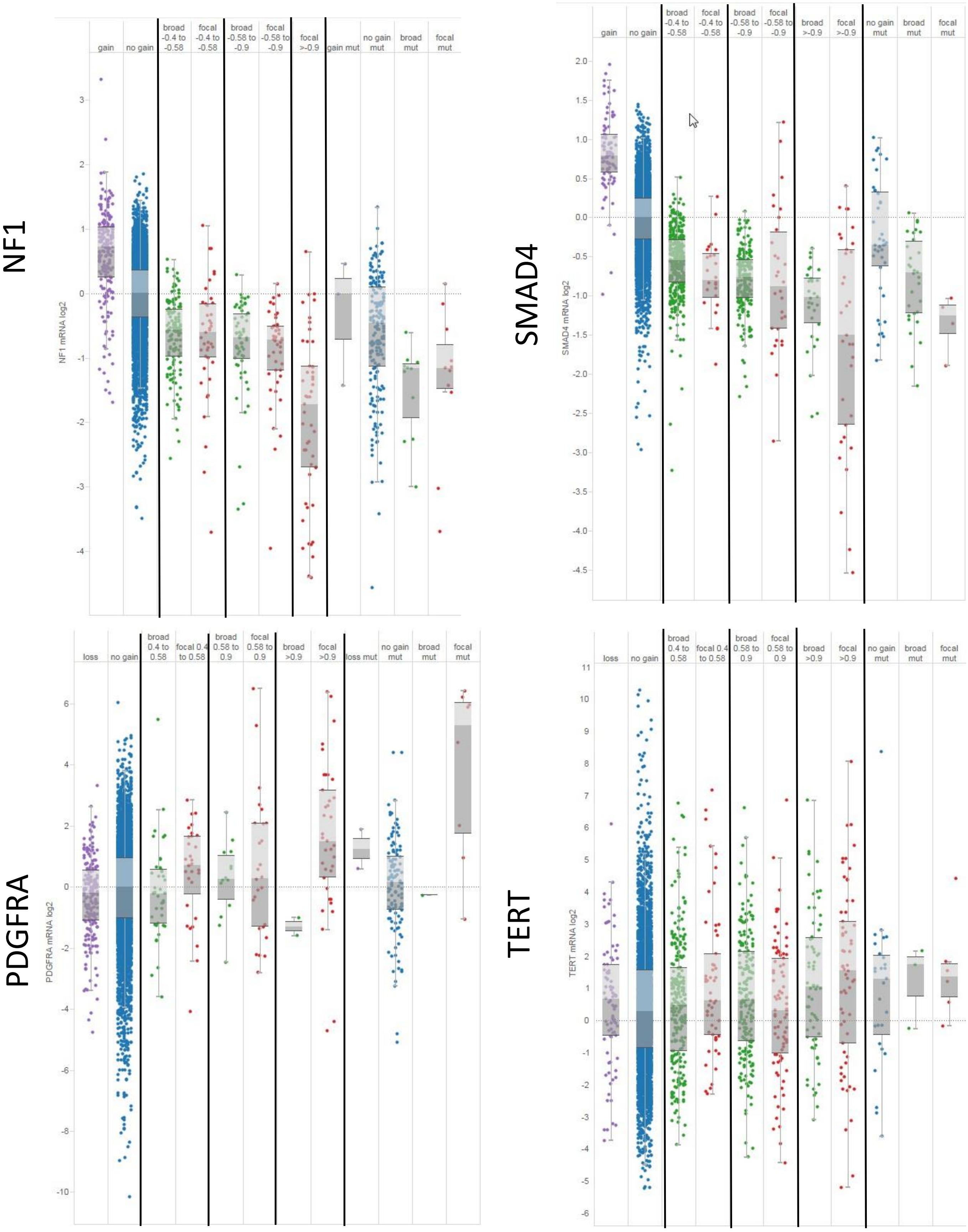

Figure S3 continued

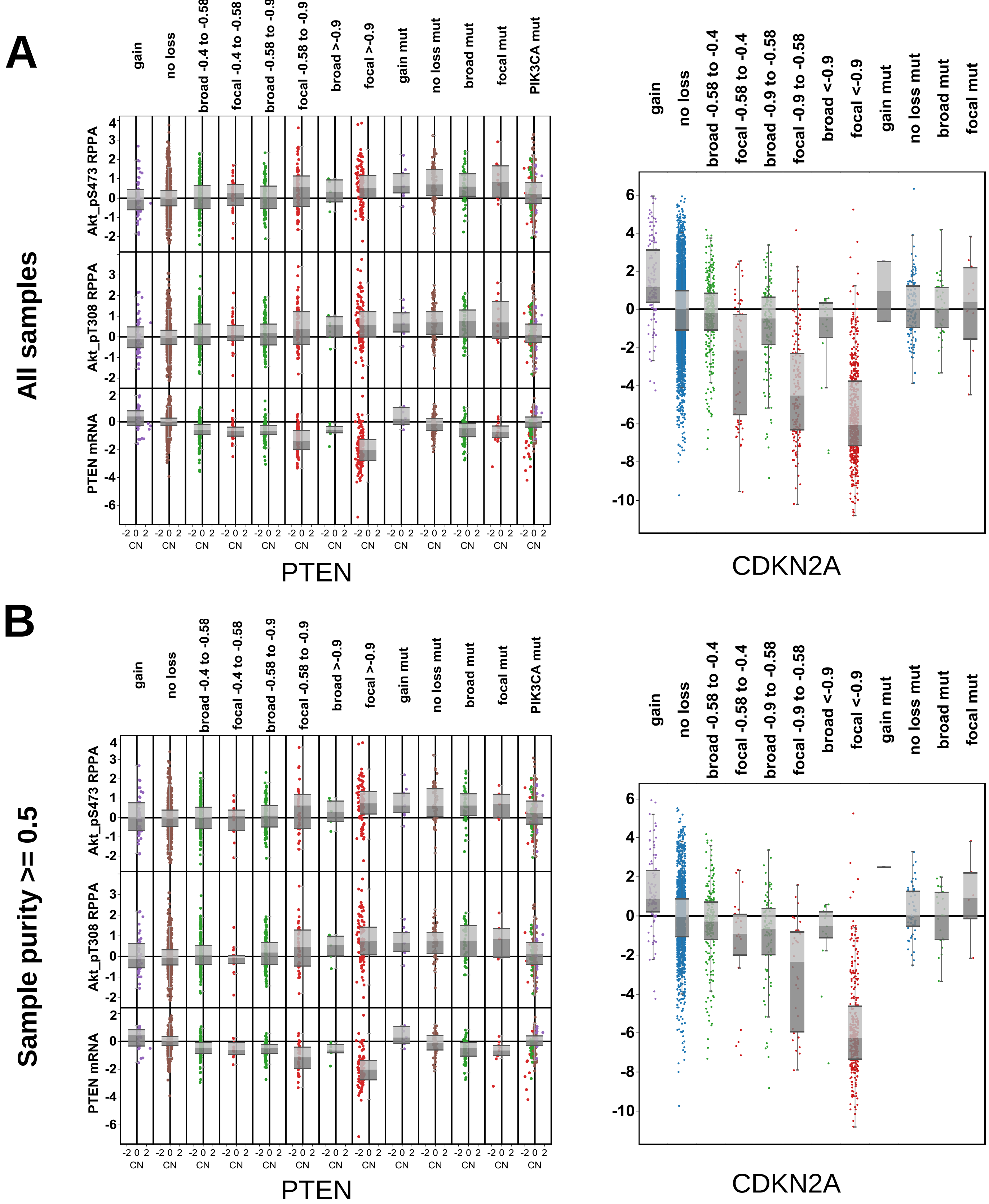

Figure S4: Effect of sample purity.
Comparing all samples (A) to samples with purity >= 0.5 (B).

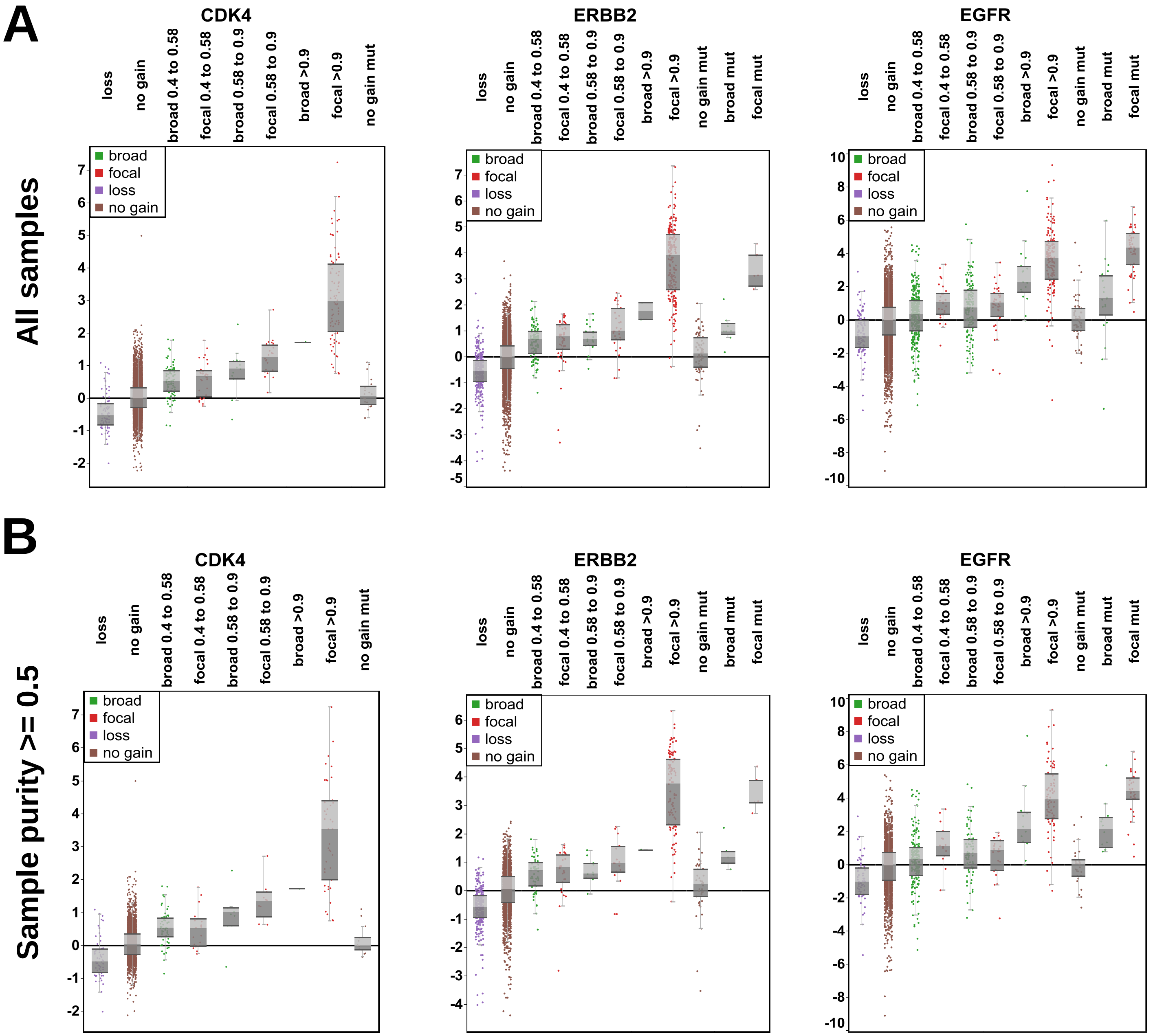

Figure S4 continued: Effect of sample purity.
Comparing all samples (A) to samples with purity >= 0.5 (B).

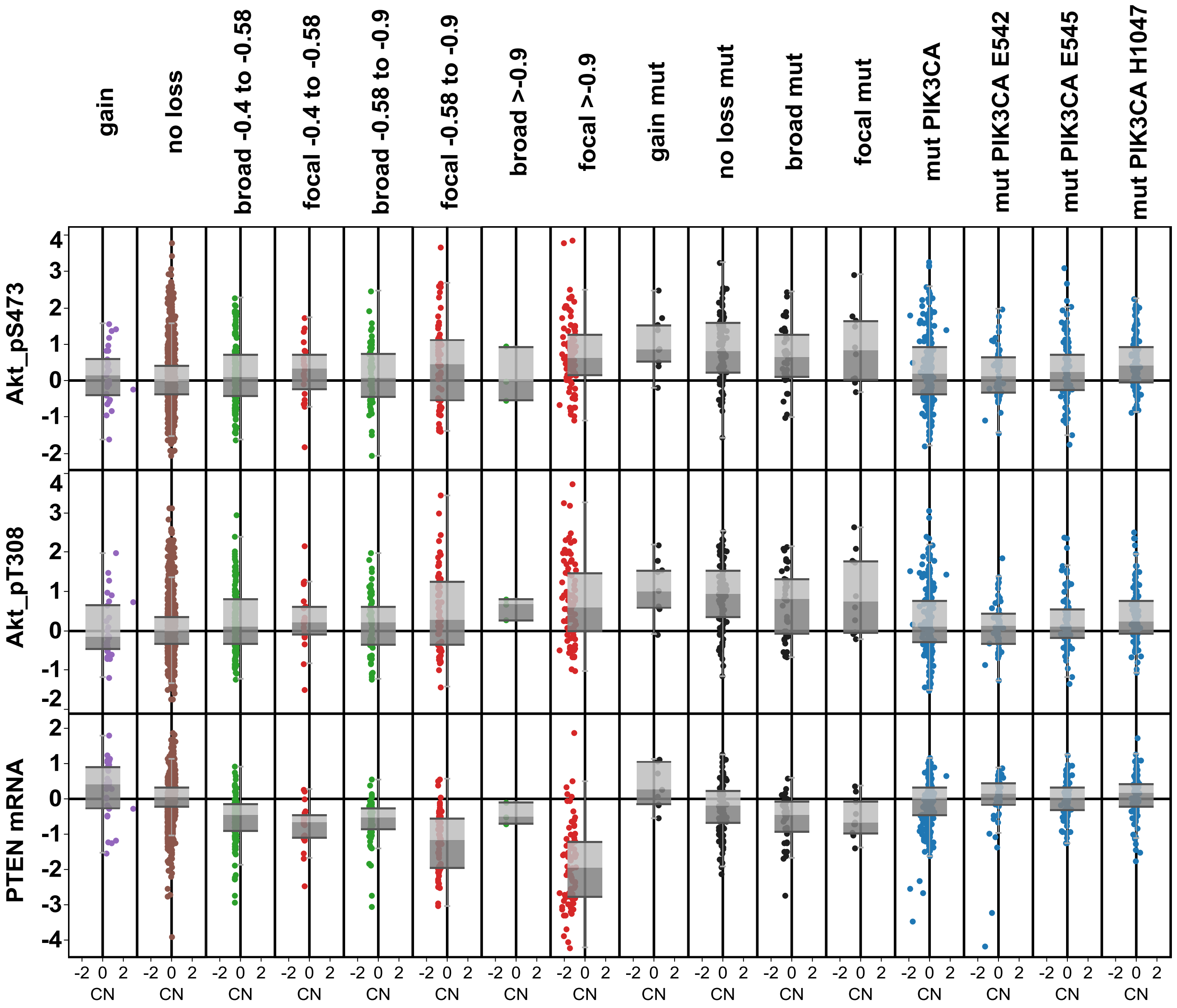

Figure S5: PTEN and PIK3CA mutation breakdowns.

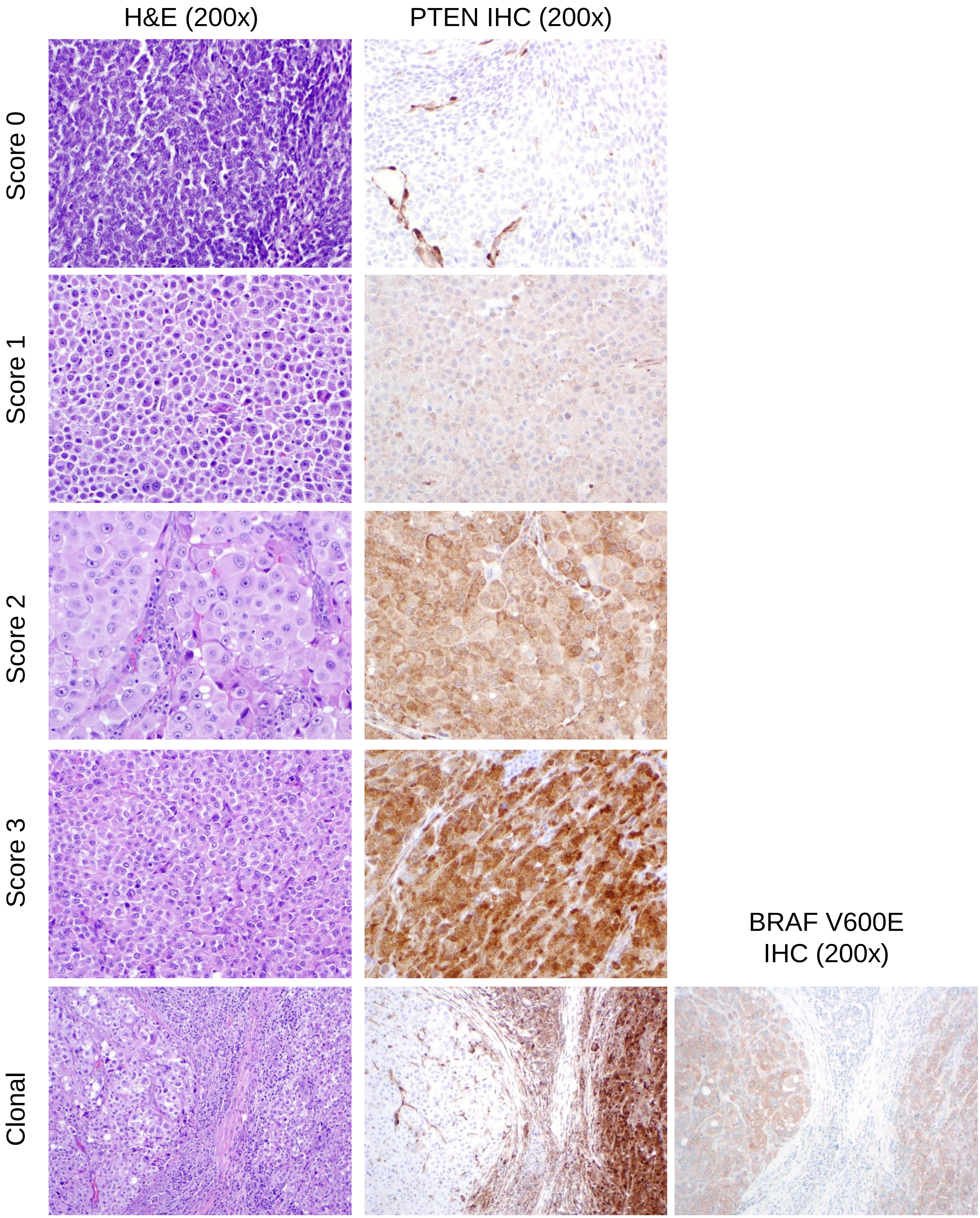

Figure S6: PTEN IHC images from TCGA patient samples.

Table S1: Cancer types and sample counts

Cancer	Samples with CN data	Samples with mRNA data	Samples with CN and mRNA data
**BLCA**	239	242	220
**BRCA**	1,029	1,154	1,025
**COADREAD**	591	262	253
**GBM**	577	166	159
**HNSC**	510	467	421
**KIRC**	504	579	498
**KIRP**	172	202	172
**LGG**	468	370	367
**LUAD**	495	548	488
**LUSC**	490	532	479
**OV**	587	265	261
**PRAD**	295	347	294
**SKCM**	376	375	374
**STAD**	331	271	236
**THCA**	502	561	500
**UCEC**	525	381	362
**Total**	7,691	6,722	6,109

Table S2: Cutoffs are stable over a range of expression thresholds. Shown are cutoffs, Youden indices, and F1 scores for length and copy number amplitude together, across all samples. Each run uses different RNA fold-change thresholds for calling a sample over- or under-expressed relative to the median of samples euploid at that locus. For example, 4/3 sets thresholds of 4-fold, above which are considered high expression changes, and 3-fold, below which are not considered high. Values in between are censored, allowing for a buffer zone. Oncogenes and tumor suppressors used different thresholds based on the standard deviation. F1 scores are only calculated for the thresholds that gave the highest Youden index. NC = no cutoff; in these cases, all cutoffs were equaled or outperformed by having no cutoff at all. N/A = not applicable due to low Youden and F1 scores.

Oncogenes	4/3 Length	4/4 Length	3.5/3 Length	3.5/3.5 Length	4/3 CN	4/4 CN	3.5/3 CN	3.5/3.5 CN	4/3 Youden	4/4 Youden	3.5/3 Youden	3.5/3.5 Youden	best F1
**CDK4**	NC	NC	NC	NC	0.9	0.9	0.9	0.9	0.909	0.866	0.910	0.883	0.914
**ERBB2**	NC	NC	NC	NC	0.95	0.95	0.95	0.95	0.893	0.861	0.880	0.868	0.935
**MDM2**	NC	NC	NC	NC	1.05	1.05	1.05	1.05	0.829	0.784	0.791	0.763	0.851
**MDM4**	NC	NC	NC	NC	0.9	0.9	0.85	0.85	0.772	0.768	0.626	0.621	0.568
**EGFR**	NC	NC	NC	NC	1.1	1.1	1.1	1.1	0.650	0.641	0.609	0.597	0.782
**CCND1**	NC	NC	NC	NC	1.1	1.1	1.1	1.1	0.564	0.510	0.527	0.494	0.756
**CCNE1**	NC	NC	NC	NC	0.85	0.85	0.85	0.75	0.452	0.428	0.421	0.416	0.669
**PDGFRA**	N/A	N/A	N/A	N/A	N/A	N/A	N/A	N/A	0.492	0.502	0.454	0.460	0.574
**MYC**	N/A	N/A	N/A	N/A	N/A	N/A	N/A	N/A	0.324	0.309	0.279	0.266	0.229
**CCND2**	N/A	N/A	N/A	N/A	N/A	N/A	N/A	N/A	0.317	0.309	0.314	0.313	0.415
**AKT3**	N/A	N/A	N/A	N/A	N/A	N/A	N/A	N/A	0.225	0.217	0.219	0.211	0.279
**TERT**	N/A	N/A	N/A	N/A	N/A	N/A	N/A	N/A	0.142	0.142	0.129	0.120	0.342
**TSGs**	**2/1.5**	**2/2**	**1.75/1.5**	**2.5/2**	**2/1.5**	**2/2**	**1.75/1.5**	**2.5/2**	**2/1.5**	**2/2**	**1.75/1.5**	**2.5/2**	**best**
**CDKN2A**	11	11	11	11	0.45	0.45	0.45	0.45	0.663	0.650	0.676	0.654	0.868
**PTEN**	15	28	28	15	0.5	0.5	0.5	0.6	0.548	0.452	0.452	0.603	0.748
**NF1**	N/A	N/A	N/A	N/A	N/A	N/A	N/A	N/A	0.301	0.266	0.256	0.384	0.542
**RB1**	N/A	N/A	N/A	N/A	N/A	N/A	N/A	N/A	0.317	0.265	0.299	0.310	0.559
**SMAD4**	N/A	N/A	N/A	NC	N/A	N/A	N/A	0.65	0.354	0.260	0.307	0.433	0.572

Table S3: 66 genes that pass the filter for amplification peaks (see Methods). The genes are partitioned into 14 contiguous DNA segments. Oncogenes selected for analysis are highlighted in green. Passenger genes selected for analysis are highlighted in yellow.

Gene	chrom	start	end	geneid	Focal Del	Focal Amp	Upstream ratio	Downstream ratio
DDAH1	1	85784168	86044046	23576	1%	3%	6.684211	8.36749
PLEKHA6	1	204187979	204329057	22874	0%	3%	1.875969	1.800169
PIK3C2B	1	204391758	204459474	5287	0%	3%	1.907592	2.073687
MDM4	1	204485507	204527248	4194	0%	3%	1.883002	2.121366
LRRN2	1	204586301	204654597	10446	0%	3%	1.642384	1.906294
ARL13B	3	93698983	93774522	200894	1%	5%	7.611098	5.245283
MECOM	3	168801287	169381563	2122	1%	3%	3.951498	1.720464
PDGFRA	4	55095264	55164412	5156	1%	3%	3.901338	2.098361
EYS	6	64429876	66417118	346007	4%	3%	2.185741	6.385502
SEC61G	7	54819940	54826939	23480	0%	5%	7.001353	3.107552
EGFR	7	55086725	55275031	1956	1%	7%	8.494665	4.150802
LANCL2	7	55433141	55501435	55915	0%	5%	5.747029	3.252053
VOPP1	7	55538306	55640200	81552	0%	3%	2.981351	2.287092
LOC442308	7	55713312	55714643	442308	0%	3%	2.695008	2.870769
FKBP9L	7	55748767	55772260	360132	0%	3%	2.620274	3.040542
SEPT14	7	55861237	55930482	346288	0%	3%	2.484211	3.134222
MRPS17	7	56019611	56023034	51373	0%	3%	2.880734	4.022691
GBAS	7	56032270	56067875	2631	0%	3%	2.803571	4.137625
PSPH	7	56078744	56119268	5723	0%	3%	2.649123	3.979499
CCT6A	7	56119378	56131682	908	0%	3%	2.474576	3.847728
POU5F1B	8	128427857	128429455	5462	0%	4%	2.044199	3.274336
LOC727677	8	128455595	128494384	727677	0%	4%	1.988403	3.292035
MYC	8	128748315	128753680	4609	0%	4%	1.941463	3.248809
PVT1	8	128902874	129113499	5820	0%	4%	1.85249	3.115044
MRGPRF	11	68771862	68780850	116535	1%	6%	2.106696	1.565919
TPCN2	11	68816350	68858072	219931	1%	7%	2.283805	1.736144
MYEOV	11	69061622	69064754	26579	1%	8%	2.725613	2.12471
CCND1	11	69455873	69469242	595	0%	8%	2.915361	2.330467
ORAOV1	11	69480331	69490165	220064	0%	9%	3.128195	2.846665
FGF19	11	69513006	69519106	9965	0%	9%	3.018423	3.216737
FGF4	11	69587797	69590171	2249	0%	9%	2.891007	3.276205
FGF3	11	69624736	69634192	2248	0%	8%	2.698631	3.203222
ANO1	11	69924408	70035651	55107	1%	9%	2.624018	3.465649
FADD	11	70049269	70053508	8772	1%	8%	2.101313	3.129524
PPFIA1	11	70116806	70230607	8500	1%	8%	2.0612	3.201603
CTTN	11	70244612	70282690	2017	1%	8%	1.835726	3.106903
SHANK2	11	70313961	70935808	22941	2%	8%	1.604725	3.073028
OS9	12	58087738	58115340	10956	0%	3%	2.950866	2.176963
TSPAN31	12	58138784	58142026	6302	0%	3%	2.520226	2.340981
CDK4	12	58141510	58146230	1019	0%	3%	2.245889	2.370803
CYP27B1	12	58156117	58160976	1594	0%	3%	1.975	2.355892
METTL1	12	58162351	58165914	4234	0%	3%	1.825399	2.296249
TSFM	12	58176528	58196639	10102	0%	3%	1.674157	2.221695
AVIL	12	58191159	58209852	10677	0%	3%	1.619574	2.176963
CTDSP2	12	58213710	58240747	10106	0%	3%	1.556632	2.117321
SLC35E3	12	69139936	69159853	55508	0%	3%	2.113328	1.667644
MDM2	12	69201971	69239320	4193	0%	3%	2.209061	1.80703
CPM	12	69244955	69357020	1368	0%	3%	2.224203	1.855072

CPSF6	12	69633317	69668138	11052	0%	3%	1.978414	1.707356
LYZ	12	69742134	69748013	4069	0%	3%	1.889254	1.695223
YEATS4	12	69753532	69784576	8089	0%	3%	1.846321	1.709317
FRS2	12	69864129	69973562	10818	0%	3%	1.658537	1.748185
PNMT	17	37824507	37826728	5409	1%	4%	2.011712	1.575085
PGAP3	17	37827375	37844310	93210	1%	5%	2.179245	1.814736
ERBB2	17	37844393	37884915	2064	1%	4%	2.050732	1.757353
C17orf37	17	37885409	37886788	84299	1%	4%	1.898172	1.825285
GRB7	17	37894162	37903538	2886	1%	4%	1.814672	1.950207
IKZF3	17	37913968	38020441	22806	1%	4%	1.765152	2.011524
ZPBP2	17	38024455	38034149	124626	1%	4%	1.568285	1.933595
VSTM2B	19	30017491	30055226	342865	1%	4%	3.577982	1.693443
POP4	19	30097170	30108162	10775	1%	4%	3.444038	1.656783
PLEKHF1	19	30156327	30166384	79156	1%	4%	3.483333	1.692308
C19orf12	19	30189793	30206696	83636	1%	4%	3.75413	1.845839
CCNE1	19	30302901	30315215	898	1%	4%	3.730143	1.861199
C19orf2	19	30414551	30507519	8725	1%	4%	3.414119	1.759906
MACROD2	20	13976146	16033842	140733	6%	3%	2.129572	2.374907

Table S4: 66 genes that pass the filter for deletion peaks (see Methods). The genes are partitioned into 39 contiguous DNA segments. Tumor suppressor genes selected for analysis are highlighted in green. Fragile site genes selected for analysis are highlighted in purple.

Gene	chrom	start	end	geneid	Focal Del	Focal Amp	Upstream ratio	Downstream ratio
CAMTA1	1	6845384	7829766	23261	4%	1%	1.912914	2.658152
LRP1B	2	140988996	142889270	53353	8%	1%	24.37592	23.75678
FHIT	3	59735036	61237133	2272	4%	0%	5.858389	5.747265
ROBO2	3	75955845	77699115	6092	3%	1%	4.073692	6.526316
KCNIP4	4	20730239	21950374	80333	3%	1%	7.348837	5.850873
FAM190A	4	91048684	92523370	401145	7%	1%	16.14536	22.4311
PDE4D	5	58264865	59783925	5144	7%	1%	3.566436	8.636101
EYS	6	64429876	66417118	346007	4%	3%	7.112416	5.082812
NKAIN2	6	124125069	125146786	154215	3%	1%	5.166235	4.748482
PARK2	6	161768590	163148834	5071	5%	1%	7.068182	2.87963
PACRG	6	163148164	163736524	135138	3%	1%	4.863636	1.981481
RPS6KA2	6	166822854	167275771	6196	3%	1%	3.684211	1.743482
CNTNAP2	7	145813453	148118090	26047	3%	1%	13.77012	7.414681
MCPH1	8	6264113	6501140	79648	3%	1%	3.075758	1.800455
SGCZ	8	13947373	15095792	137868	4%	1%	3.533835	3.627438
NRG1	8	31496820	32622558	3084	3%	2%	1.875932	1.959596
UNC5D	8	35092975	35652181	137970	3%	3%	1.638916	1.763076
PTPRD	9	8314246	10612723	5789	8%	1%	3.678231	4.485169
IFNA5	9	21304613	21305312	3442	7%	1%	3.365854	1.688124
KLHL9	9	21331018	21335429	55958	8%	1%	3.138264	1.895146
IFNA6	9	21350317	21350886	3443	8%	1%	2.949254	1.960317
IFNA2	9	21384254	21385396	3440	8%	0%	2.244237	2.120506
IFNA8	9	21409146	21410184	3445	8%	0%	2.165128	2.499058
IFNA1	9	21440453	21441315	3439	8%	0%	2.007898	2.915363
MTAP	9	21802635	21865970	4507	12%	0%	2.770769	5.38954
C9orf53	9	21967138	21967753	51198	15%	0%	3.31566	6.669579
CDKN2A	9	21967751	21994490	1029	16%	0%	3.489035	7.523231
CDKN2BAS	9	21994790	22121096	1E+08	12%	0%	2.417497	6.063124
CDKN2B	9	22002902	22009312	1030	15%	0%	3.043885	8.862222
PAX5	9	36838531	37034476	5079	4%	2%	3.870968	5.603448
PRKG1	10	52750911	54058110	5592	3%	1%	3.822222	5.117835
NRG3	10	83635070	84746935	10718	3%	1%	5.831909	1.683673
PAPSS2	10	89419476	89507462	9060	4%	0%	2.357566	1.860656
ATAD1	10	89512875	89577917	84896	4%	0%	2.464865	2.216871
CFLP1	10	89578070	89605369	142913	4%	0%	2.520542	2.426131
KILLIN	10	89618918	89623194	1E+08	4%	0%	2.606419	2.532903
PTEN	10	89623195	89728532	5728	6%	0%	3.805093	3.809524
RNLS	10	90033621	90343082	55328	3%	0%	2.091837	2.181667
LIPJ	10	90346519	90366733	142910	3%	0%	1.591837	1.733333
LIPF	10	90424146	90438572	8513	3%	0%	1.59067	1.791789
DLG2	11	83166055	85338314	1740	4%	2%	5.753086	5.614458
CNTN5	11	98891706	100229616	53942	3%	1%	4.453275	6.555023
SOX5	12	23685231	24715383	6660	3%	2%	4.631579	4.07572
RB1	13	48877883	49056026	5925	3%	0%	3.118577	3.333417
NPAS3	14	33408459	34273382	64067	3%	2%	2.08284	5.866667
GABRG3	15	27216429	27778373	2567	3%	1%	1.714462	2.785278
A2BP1	16	6069132	7763340	54715	7%	2%	4.13529	15.10807

WWOX	16	78133327	79246564	51741	7%	1%	9.659574	4.022308
CDH13	16	82660399	83830215	1012	7%	1%	6.475664	3.107637
NF1	17	29421945	29704695	4763	3%	1%	1.798825	1.597577
ACCN1	17	31340105	32483825	40	4%	2%	1.962518	1.800813
SMAD4	18	48556583	48611412	4089	3%	0%	2.621995	2.242991
SMARCA4	19	11071598	11172958	6597	3%	2%	2.83871	2.461538
ZNF429	19	21688437	21721079	353088	3%	1%	2.465734	3.75368
MACROD2	20	13976146	16033842	140733	6%	3%	8.873279	7.432561
C21orf81	21	15316090	15352765	391267	3%	1%	7.486191	1.698689
LIPI	21	15481134	15579254	149998	3%	1%	6.96885	1.890055
ABCC13	21	15646120	15673707	150000	3%	1%	6.335655	2.568089
SAMSN1	21	15857549	15955723	64092	3%	1%	6.456803	2.709677
USP25	21	17102496	17252377	29761	3%	1%	6.99487	3.096742
C21orf34	21	17442842	17982094	388815	3%	2%	7.591307	3.373914
NCAM2	21	22370633	22912517	4685	3%	1%	2.942465	3.545849
DSCAM	21	41384343	42219039	1826	3%	1%	2.791865	1.81017
TMPRSS2	21	42836478	42880085	7113	4%	1%	1.988217	2.413525
LARGE	22	33669062	34316416	9215	3%	1%	2.502313	2.426815
DMD	X	31137345	33357726	1756	4%	1%	9.799245	7.858049
